# Experiences and Needs of Multicultural Youth and Their Mentors, and Implications for Digital Mentoring Platforms: Qualitative Exploratory Study

**DOI:** 10.2196/15500

**Published:** 2020-02-04

**Authors:** Rebecca Lynn Radlick, Jelena Mirkovic, Sarah Przedpelska, Elanor Halvorsen Brendmo, Deede Gammon

**Affiliations:** 1 Norwegian Research Centre (NORCE) Bergen Norway; 2 Center for Shared Decision-Making and Collaborative Care Research Oslo University Hospital Oslo Norway; 3 Catalysts Association Oslo Norway; 4 Norwegian Centre for E-health Research University Hospital of North Norway Tromsø Norway

**Keywords:** e-mentoring, immigrants, social capital, youth, mentoring, eHealth

## Abstract

**Background:**

Mentoring programs (ie, programs that connect youths with adult volunteers) have been shown to improve outcomes across the behavioral, social, and academic domains of youth development. As in other European countries, mentoring programs have few traditions in Norway, where interventions for multicultural youths are usually profession driven and publicly funded. Faced with the risk of disparities in education and health, there is a need to better understand this group’s experiences and requirements relative to mentoring. This would also serve as a basis for designing and implementing digital support.

**Objective:**

The objective of this study was to gain insight into multicultural youth mentees’ and adult mentors’ experiences and needs in the context of an ongoing mentoring program, how digital support (electronic mentoring) might address these needs, and how such support could be designed and implemented.

**Methods:**

The study used a qualitative approach, with data from 28 respondents (21 mentees and 7 mentors). In total, 4 workshops with mentees as well as semistructured interviews with mentees and mentors were conducted. The sessions were audio recorded, transcribed, and analyzed thematically.

**Results:**

In total, 3 main themes were identified from the experiences and needs reported by the mentees and mentors. These included a need for connection, help in achieving goals, and the need for security and control. Subthemes encompassed a desire to socialize with others, balancing the nature of the relationship, paying it forward, building trust, sharing insights and information with peers, goal-oriented mentees and mentors wanting to assist with goal achievement, and the fundamental need for privacy and anonymity in the digital platform.

**Conclusions:**

The findings of this study are supported by the literature on traditional mentoring, while also offering suggestions for the design of digital solutions to supplement the in-person mentoring of multicultural youth. Suggestions include digital support for managing the mentee-mentor relationships, fostering social capital, and ways of ensuring security and control. Features of existing electronic health apps can be readily adapted to a mentoring program context, potentially boosting the reach and benefits of mentoring.

## Introduction

### Background

School dropout among adolescents and young adults has been increasingly reframed as a public health issue in light of the strong association between poor self-rated health in adolescence, high school dropout, and reduced labor market integration [1-3]. Immigrant youths in Norway are at a greater risk for unemployment or leaving school early and exhibit a 26-percentage point disparity in education and employment, compared with native Norwegian youths [4]. Factors contributing to these disparities within the immigrant youth population include time spent in Norway and, thus, language abilities, reason for migration (refugee vs labor migration), health status, and parental income [4].

In light of the aforementioned discrepancy in school and labor market participation across immigrant and majority youth, there is an increased willingness to try out alternative models, such as mentoring programs, to reduce these disparities. Mainly studied in the United States, mentoring is defined as “taking place between young persons (ie, mentees) and older or more experienced volunteers (ie, mentors) who are acting in a nonprofessional helping capacity to provide relationship-based support that benefits one or more areas of the mentee’s development” [5]. A 2001 meta-analysis of 73 independent evaluations of mentoring programs supports the effectiveness of mentoring in improving outcomes across behavioral, social, and academic domains [6], as does a more recent meta-analysis of outcome studies [7]. However, mentoring programs, which are typically run as social entrepreneurships, have few traditions in Norway, where interventions for immigrant youths are often profession driven and publicly funded [8].

One of the first mentoring programs in Norway (called Catalysts) targets recently arrived immigrant youths aged between 16 and 25 years. The majority are recruited through introductory language classes at their schools, which are mandatory for everyone who wishes to complete high school in Norway. Participants’ immigration background varies and includes unaccompanied minor refugees, family reunification, and children of labor migrants. The Catalysts mentoring program lasts for 6 months and matches immigrant youths (mentees) and volunteer adults (mentors) in mentor-mentee dyads according to their interests and needs. Program components are closely aligned with mentoring best practices [5] and build on the principles of appreciative inquiry [9,10], closely related to positive psychology [11]. The Catalysts program is primarily commissioned by municipalities and corporate social responsibility entities in businesses, in addition to funding from the Norwegian Labor and Welfare Administration (NAV).

### Electronic Health and Adaptations to a Mentoring Context

The researchers who initiated collaboration with Catalysts have backgrounds in electronic health (eHealth) and sought ways of applying eHealth knowledge and apps to health promotion interventions outside of health care settings for health promotional purposes. Catalysts mentoring practitioners, for their part, were interested in evidence-informed digital innovations to improve the reach and effectiveness of their programs. We joined forces to illuminate the following overarching question: to what extent could an evidence-informed electronic mental health platform (called ReConnect, see also the Methods section) be adapted to the needs and experiences of mentees and mentors to enhance mentoring? As described elsewhere [12,13], the original ReConnect platform was designed for individuals requiring long-term mental health care. It included 3 main components: a peer support forum, a secure messaging function between service users and their health care providers, and a toolbox of resources that could be used autonomously or in collaboration between service users and providers (with resources related to mapping strengths, mindfulness, sleep hygiene, goal-setting modules, personal network, and medications). The design and operation of ReConnect were guided by principles of recovery [14], which may or may not resonate with stakeholders of youth mentoring.

Little prior research was available, as only 3% of mentoring programs in the United States have a digital component and only 1% are exclusively digital [15]. The few implementations of digital components previously studied range from informal and supplemental to more formal or exclusive (digital interactions only) and include email, social media, and SMS as well as app-mediated connections and computer platforms [16]. Research indicates that demographic and personal circumstances, communication styles, accessibility issues, and program implementation shape the effectiveness of electronic mentoring (e-mentoring) among youth, but many questions remain about what types of digital solutions may be effective and for whom [16].

### Theory of Change

To assess the relevance of the pre-existing eHealth platform to the context of mentoring and to identify necessary and desired adaptations, we encouraged Catalysts to articulate their program’s *theory of change*. Along with many in the mentoring field [17], Catalysts maintains that in-person relationships between mentees and mentors are foundational and that any digitalization should aim to enhance, not replace, in-person relationships. In addition to appreciative inquiry [10], which explicitly redirects attention away from problems and vulnerabilities toward strengths and opportunities, Catalysts’ theory of change encompassed elements closely aligned with social capital [18-22], particularly the *bridging* type of social capital that is found across the lines of age, social status, and ethnicity [23]. Evidence suggests that having wider support networks of peers and unrelated adults across a range of domains (that is to say, greater social capital) is associated with a variety of positive outcomes, including better youth mental health [24,25]. These perspectives might broadly be seen as complementary to the recovery-oriented perspectives and literature that guided the design of the original ReConnect platform [12]. Nevertheless, the efforts to arrive at clearer (theoretical) rationales for how to adapt ReConnect and why (or instead start from scratch) called for the perspectives of mentees and mentors. To elicit these perspectives, an open inductive approach to e-mentoring was considered most appropriate at this stage.

### Research Questions

This exploratory study aimed to gain insights into the experiences and needs of immigrant youths and their mentors and how the mentoring experience might be enhanced by a digital supplement. More specifically, the following questions guided the study:

What are the needs of mentoring stakeholders (mentors and mentees)?

How can these needs be addressed using a digital platform?

How can such a platform be designed and implemented within this mentoring context?

Insights into these issues will be used as a basis for subsequent formulations of user requirements of an e-mentoring platform, and thereafter an intervention study, to assess the effects of the platform.

## Methods

### Study Design

The study was conducted between winter 2018 and spring 2019 and was approved by the Data Protection Officer at Oslo University Hospital. Qualitative data were collected with different methods to illuminate the research questions, in close collaboration with stakeholders (mentees and mentors). As outlined in Table 1, we conducted interviews, focus groups, and workshops at the Center for Shared Decision Making and Collaborative Care Research (Oslo University Hospital), Norwegian Research Center (NORCE), and Catalysts localities to gain insights from stakeholders about their needs and preferences for digital support to augment their face-to-face mentoring meetings.

### Participant Recruitment

Mentors and mentees were recruited from among current or previous participants in the Catalysts program in several ways: an open call for participation among active mentors and mentees or by direct contact from the program coordinator**.** In the latter case, a purposive sampling of mentors and mentees was used to select participants with varied backgrounds in terms of program progression and demographic characteristics [26]. Each person from this list of mentees was then contacted (by EB or SP), and interviews or workshops were scheduled. All participants received information about the purpose of the study, the voluntary nature of participating, and maintenance of confidentiality, and all participants signed informed consent forms. The interviews and focus group workshops were audio taped and transcribed, with the exception of the pair interview, which was not taped because of technical problems. There were 28 different respondents, including 21 mentees and 7 mentors (Table 1).

**Table 1:** Data collection method and respondent roles.

Data collection approach	Respondents’ role (number of respondents)
Interview with mentees	Mentee (n=2)
Interview with mentor	Mentor (n=6)
Interview with mentor and mentee pair	Mentor (n=1) and mentee (n=1)
Focus group workshops (n=4) with mentees	Mentees (n=23; 18 unique); 5 participated in multiple workshops

### Data Collection

Participants were interviewed in 8 individual interviews (2 mentees and 6 mentors) and 1 pair interview (1 mentor and 1 mentee), as illustrated in Table 1. Mentees were interviewed in person, whereas mentors were interviewed both in person and via telephone. The individual interviews were conducted by 2 of the authors (EB and RLR) and lasted between 30 min and 1 hour. A total of 4 focus group workshops were also conducted, with 18 different mentees participating; an additional 5 mentees participated in multiple workshops. Here, an author (SP) acted as a moderator and guided the activities and conversations.

Mentees were interviewed at different phases of the program, allowing us to gain insights into the youths’ expectations, experiences, and needs as they progressed in the program. All mentors were interviewed toward the end of the program or after the programs had concluded. A semistructured interview guide was used, and the initial questions posed to respondents were intentionally broad, addressing expectations and motivations for participation; challenges and benefits of the program; and, specifically, the use of apps. Over time, as the requirements for the design of an e-mentoring platform began to take shape, questions became more specific, for example, “If an app were to be introduced as part of the program, what would be useful to you to have in such an app, and why?” During the discussions, participants were asked follow-up questions to expand on what was said and to reflect upon their answers if needed [26]. Respondents were also encouraged to share their concrete experiences in participating in the mentoring program, elaborating on how digital support might strengthen or undermine specific aspects of the program. Workshops allowed interactions among participants, who could mutually confirm, reinforce, and contradict each other’s statements, whereas the interviews allowed deeper probing around the functionality of the mentor program and how it could be improved with a digital innovation. In the workshops and interviews, the original ReConnect platform (described earlier), which included 3 interactive components (ie, peer support forums for users and health care providers, a toolbox of resources, and secure messaging), was used as a point of departure. Features and design principles from ReConnect were mentioned when posing questions about its relevance to participants’ needs and preferences in a mentoring context. Given the ReConnect platform’s medical content, archaic looks, and code libraries, the study group decided to use the features and design principles in developing a new, but similar, platform. In the workshops, participants engaged with hand-drawn wireframes based on some of the features from ReConnect, such as the forum, messaging, and toolbox. Stakeholders were asked how they would engage with the aforementioned features in a mentoring context, to assess their needs. This selection of features from the ReConnect platform potentially influenced respondents’ feedback on specific possibilities for an e-mentoring platform.

### Data Analysis

Data were analyzed using thematic analysis, where the researchers first familiarize themselves with the data; generate initial codes; assess, review, and name themes; and then report findings [27]. The recordings from the interviews and workshops were transcribed and imported to NVivo (QSR International Pvt Ltd) qualitative data analysis software. In the first step of the analysis, 3 authors (DG, EB, and SP) independently read the transcriptions of the interviews and workshops to get an overview of topics reported by the participants and to generate initial codes from the transcripts. The code generation and analysis were completed with the initial research questions in mind, focusing on the needs of the respondents, how an e-mentoring platform could address such requirements, and how to design and implement the platform. All manifest content, or descriptions of needs, experiences, and ideas that were considered relevant to creating, adapting, and implementing the e-mentoring platform, were extracted from the transcriptions. The initial codes were generated, compared, and discussed between the 3 authors. The fourth author (JM), who is a computer design scientist, observed several of the workshops and participated in discussions of emerging codes in light of design implications. Overlapping or similar meaning codes were grouped together into initial themes. The themes were reviewed and discussed iteratively by 4 of the authors (DG, EB, SP, and JM), with the aim to reduce the number of themes and define meaningful and relevant names of the themes without losing relevant meaning. After defining codes and themes, the fifth author (RLR) then independently read and coded the material, comparing with already extracted codes. All authors discussed the results to reach consensus on final names of the themes and codes for subthemes. The Norwegian quotations were translated by RLR and DG, who are both native English speakers and fluent in Norwegian.

## Results

### Overview

**Figure 1:**
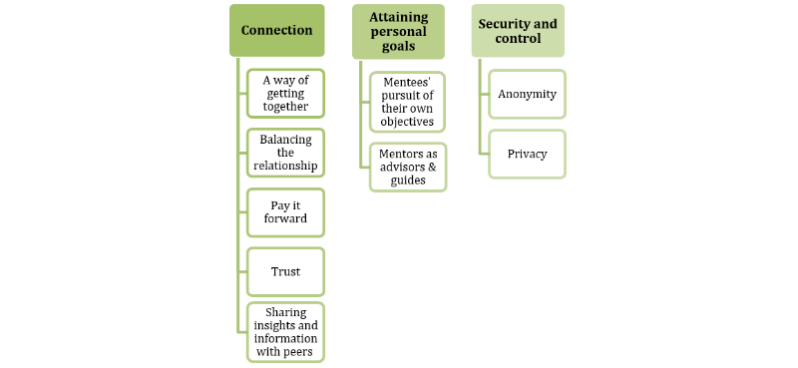
The main themes and subthemes.

A total of 3 overarching themes were identified in the respondents’ descriptions of the needs and preferences for traditional mentoring. The main themes identified were related to connection, personal goals, and security and control. Most subthemes were common to both mentees and mentors, but several subthemes were more closely related to a single respondent group. Each of the overarching themes and subthemes are first presented, followed by a summary of suggestions from the informants about how digital support might address such needs. Quotations that are particularly illustrative of the theme are included in the text, whereas supplementary quotations are referenced and are available in Multimedia Appendix 1. A summary visualization of the themes and subthemes is depicted in Figure 1.

### Theme 1: Connection—A Sense of Community

The first overarching theme identified in the data was the stakeholders’ desire for connection or belonging to a larger community. This concept was reflected in mentor-mentee relationships, connections within the separate mentee and mentor spheres, and links to Norwegian society. A mentee described challenges with regard to the latter point:

You know Norwegians, if you don’t speak to them first, they won’t speak with you.

Mentee, Interview 2

Subthemes extracted from the transcripts included a desire to get together with others, balancing the relationship, paying it forward, trust, and the need for sharing insights and information with peers.

#### A Way of Getting Together

The first subtheme encompassed the need for group activities and ways of socializing. A core argument for introducing a digital platform as part of the program was to enhance contact between the mentee-mentor dyads. Many of the mentees expressed a desire for frequent contact with their mentors, as illustrated by the following mentee quotation (when asked how often she would like to meet her mentor):

If possible five days a week! Every day!

Mentee, Interview 1

The youths emphasized that an app should not be used at the expense of human contact (meetings with mentors) but rather supplement it. Mentees already felt that the in-person contact was too infrequent (Multimedia Appendix 1) and that emotional bonding, showing empathy, and closeness could be challenging to facilitate remotely and would be preferable in person (Multimedia Appendix 1). However, the same mentee acknowledged that context was important, and for practical urgent needs, such as feedback on a job application, face-to-face contact was less essential (Multimedia Appendix 1).

In addition, multiple mentees expressed a desire to find events and arrangements locally and saw the program as a way to do so. For example, although one of the mentees wanted to be more socially active, before participating in the program, she usually just went home and stayed alone (Multimedia Appendix 1). The need to meet other people and participate in activities was also apparent in this quote from one of the youths describing his town:

I hear those who live in [a city], they have lots of activities and stuff…And then, there’s [a rural place] where I live. I see more trees than people [laughs]. And in the wintertime there’s no people. Only sometimes I meet an old lady and an old man, walking their dog.

Mentee, Workshop 1

For the adults, the social aspect of meeting other mentors with different backgrounds was mentioned as a motivation for volunteering. Some reported having expanded their own networks of contacts by meeting other mentors (Multimedia Appendix 1). Similar to the youth, mentors also emphasized the importance of in-person contact with their mentees (Multimedia Appendix 1).

#### Balancing the Nature of the Relationship

A second subtheme relating to the need for connection addressed the nature of the mentor-mentee relationship and balancing relationship roles. For example, several of the mentors experienced blurred boundaries between being a mentor (formal role) and being a friend (informal role). For some mentors, the blurring of the roles was quite natural (Multimedia Appendix 1) and could develop organically (Multimedia Appendix 1), whereas for others, it was a conscious choice:

I have seen this as more of a friend relation...so I have chosen to not be so formal because…it’s another person. This isn’t some [social work case in the public system].

Mentor, Interview 4

This more informal relationship was echoed by a mentor who underscored the spontaneity of interactions with their mentee (Multimedia Appendix 1). Although many of the mentors described permeable boundaries between the formal and informal, they also expressed concerns about *intruding* on their mentees’ personal spheres when using social media for communicating.

#### Pay it Forward

Both mentees and mentors articulated a need to share their knowledge and experiences—to *pay it forward.* The youths felt that they benefited from having a mentor, and several of them described wanting to share their experiences and knowledge with their fellow mentees, future mentees, or other youths who were not fortunate enough to have a mentor (Multimedia Appendix 1).

Mentors also mentioned the desire to pay it forward as one reason for volunteering in the program. For example, some had mentors of their own at work or had prestigious jobs, and some felt a responsibility for sharing their knowledge and experience, in helping with integration in Norway (Multimedia Appendix 1) or in trying to help as many people as possible (Multimedia Appendix 1). Other mentors felt motivated by the possibility to share their own experiences of being new to a company, city, or country or by having been in similar circumstances as the mentee. These mentors recalled what it felt like to be young and uncertain of what direction to take in life or how to get there, and they felt a strong desire to help (Multimedia Appendix 1). Mentors also recognized the effect the relationship with mentees had on their own personal development and that they learned a lot not only about other people but also about themselves (Multimedia Appendix 1). However, this expectation of *paying it forward* sometimes conflicted with reality, as experienced by a mentor whose mentee had a broad network and significant resources before joining the program:

I learned a lot about the daily life of youths in his situation, and the situation he has been in. I would have liked to have helped him more, but in a way, that is “egotistical altruism” or whatever you want to call it. That you wish you could do more. But it is of course only a good thing that he didn’t have a need for more.

Mentor, Interview 5

#### Relational Trust: A Supportive and Safe Environment

The mentees highlighted trust as important, but challenging, in their relationship with mentors. This was partially attributed to the (perceived) brevity of the program (6 months). Talking about personal issues requires trust; however, trust takes time to develop. However, the requirement for mentors to sign confidentiality agreements ameliorated this issue for some mentees (Multimedia Appendix 1). There was also a discussion on whether it would be preferable to meet face-to-face first or via an app. Several mentees suggested that it would be preferable to meet in person first before engaging in any kind of digital interaction, as they only interact with people online when they actually know the person and have built trust with them (Multimedia Appendix 1). However, others opined that using an app first could be useful for getting to know each other before the first meeting. The fact that only authorized users could have access to the platform (as discussed in the section below on security and control) could also help enhance the feeling that it is a safe environment to share personal information. Trust was also discussed by the mentors as fundamental in developing the relationship with their mentee (Multimedia Appendix 1).

#### Sharing Insights and Information With Peers

Both mentors and mentees expressed a need for information and insights in navigating their relationships, particularly through discussion with others in the same role. Several of the mentees wanted to talk with other youths, without mentors being present:

Because this was the first time I had a mentor in my life. Maybe it’s the same for the others. So it can be a bit challenging to navigate, at least the first time. So we share tips between us, and meet up, and similar.

Mentee, Workshop 1

The adults were also new to mentoring and wanted more information on best practices and specific scenarios that they might encounter. A mentor discussed how she needed to give her mentee information on how to apply for study grants, as well as about citizenship requirements, but that this information was not readily available and was time-consuming to find (Multimedia Appendix 1). Another mentor mentioned that the communication between the group of mentors at meetings was quite good, but that there was no communication outside of the meetings, and that supplementary contact was desirable (Multimedia Appendix 1). Some mentors expressed uncertainty as to what they should do at the in-person meetings with their mentees and wanted additional guidelines, potentially to ensure that they were maintaining standards of best practice. However, although many of the respondents shared a desire for peer-to-peer support, some of the mentors and mentees did not find support and contact with other adults or youths, respectively, as something necessary.

### Theme 2: Attaining Personal Goals

A second overarching theme emerging from the data was related to goals and expectations and was reflected in both mentor and mentee responses. The mentees were motivated and had clear ideas of what they needed support with, whereas the mentors frequently viewed themselves as guides, encouraging and helping their mentees to achieve these goals.

#### Mentees’ Pursuit of Their Own Objectives

The youths overwhelmingly viewed the program as a way to help them in attaining their goals, and many sought support for broad needs related to education and career (Multimedia Appendix 1). Mentees were sometimes very specific about their needs for assistance with homework and school; learning better computer skills; or improving their Norwegian, both formal and colloquial:

I would really like advice on what’s best for me. For example, what I’ll do in the future. What career I should choose, or how I can be integrated into Norwegian society, and better understand Norwegian culture. I’m going to live in Norway, right? …I really want to learn Norwegian [slang], like [young people speak] on the streets.

Mentee, Workshop 1

A number of the mentees mentioned strengthening or broadening their networks as another key motivation for participating in the Catalysts program, and this opportunity to meet a supportive nonrelative adult was appealing (Multimedia Appendix 1).

#### Mentors as Advisors and Guides

Mentors reported seeing themselves as guides, advisors, or *coaches*. Many mentors viewed one aspect of their work as supporting their mentee in achieving specific objectives, such as finding a job, as well as motivating and following up on the mentee’s progress (Multimedia Appendix 1).

One mentor helped her mentee get a part-time job at a customer service center after the mentee expressed an interest in a job where she could help other people (Multimedia Appendix 1). At the same time, some mentors expressed uncertainty about their roles when faced with highly motivated mentees who had already identified career objectives that they appeared well equipped to pursue. Sometimes these mentees were able to ask for and receive help from others in their existing network. This led to some frustration from one of the mentors who clearly had a strong desire to help with specific tasks:

I think it was a great experience [mentoring], but also a little frustrating. I felt that my mentee didn’t really have much of a need for a mentor. We struggled with finding things to work with, since he had connections with other adults that he was in contact with regularly, who he could ask the same questions to...He had a better, more well-established network around him rather than just him and me...

Mentor, Interview 5

### Theme 3: Security and Control

The respondents expressed a range of needs that had to do with security and control over their personal information. A main reference point was social media (such as Facebook and Instagram), and many of the mentees experienced these platforms as being unsafe (Multimedia Appendix 1). *Anonymity* was a subtheme identified in the data, although both mentors and mentees were not in complete agreement on whether anonymity was desirable or not. The second subtheme was a concern about *privacy*. The need for security and control was discussed in light of particular e-mentoring platform features (as presented in the subsequent section): a forum, messaging, and toolbox.

#### Anonymity

Some mentees wanted to remain anonymous in the forum all or some of the time, whereas others were uninterested in the option; their reasoning was often context dependent. Several youths wanted very personal discussions related to mental health to be private, whereas less sensitive topics such as choosing school courses or dealing with parents were acceptable for nonanonymous discussion with mentors and other mentees. Those supporting anonymity described how they might want to share sensitive issues without concerns of being identified. These respondents felt that it could be useful to present problems anonymously and to discuss them, both with other mentees, in case of having misunderstandings with the mentor, and with the mentor later, if it was a personal issue (Multimedia Appendix 1). Those opposed to the option of anonymity expressed concerns that it might undermine the networking and connective potentials of the forum as well as present a hindrance in building trust between participants (also discussed in the first section on *connection*; Multimedia Appendix 1). One mentee suggested that it is quite difficult to trust, build connections, and discuss personal topics if some people are anonymous, whereas others are not:

But [being] anonymous, it is [a] little bit…maybe someone will not talk with you. They will think, ok, she is anonymous...I don’t know [who she is]...and...don’t want to put her information. So, with who I’m talking. It is, like, difficult to trust.

Mentee, Workshop 3

Mentors expressed concerns for respecting their mentees’ private spheres and discomfort in mixing private social media usage with mentoring. This also relates closely to distinguishing between more formal and informal roles (as discussed in the section on balancing the nature of the relationship).

#### Privacy

The second subtheme, *privacy*, relates to who has access to personal information. Mentors and mentees expressed needs for limiting unauthorized access and being able to trust that people are who they say they are in the platform. Ensuring that mentee data would not be leaked or that unauthorized people would not gain access was described as being critical. Similarly, the mentors articulated strong concerns about their mentees’ privacy and maintaining confidentiality. Privacy was mentioned by mentees relative to family members who could sometimes be intrusive; this becomes more poignant when one considers the potential for social control. As a female mentee expressed:

I have a brother, and he said to me, “you aren’t allowed to talk with [this] boy”...my father knows...but [my brother] thinks this way because he doesn’t like that I talk with boys or hang out with boys.

Mentee, Interview 2

Cultural differences between countries of origin and Norway with regard to privacy were mentioned by another mentee who appreciated high-level security in logging on and how it prevented others from gaining access to private information (Multimedia Appendix 1).

### Digital Support for Identified Needs

The youths and mentors mentioned numerous experiences and needs, as outlined above, and suggested multiple ways that digital support could help meet these needs. Throughout these discussions, various elements from the ReConnect platform [12] were presented as examples of what might be possible in the e-mentoring platform. These included a *forum*, *messaging function*, and *toolbox*. The youths also proposed additional potential features in the e-mentoring platform including GPS and an activity *calendar*.

#### Forum

Both mentees and mentors identified multiple purposes for *forums*. Forums were suggested as a way for mentees to pay it forward, sharing knowledge they had gained through the mentoring experience with other youths in the program, with the next group, or with youths not in the program (Multimedia Appendix 1). Initiating and discussing issues anonymously was another use for a forum, as seen by the mentees. A group mentor-mentee forum could allow connections between mentors and mentees with similar interests or mentees who needed advice on a particular topic or career, allowing better maximization of mentor resources, as suggested by both youths and mentors (Multimedia Appendix 1).

A mentor-only forum was mentioned by the mentors as potentially providing a place where they could initiate conversations with other mentors, becoming more secure in their role, and increasing feelings of connection to the mentor group as a whole. Being able to ask for advice and best practices in a forum was mentioned by many of the mentors (Multimedia Appendix 1). However, one mentor felt that concrete and simple issues could be discussed in an electronic forum but questioned the extent to which complex issues could be discussed this way without losing important context (Multimedia Appendix 1).

In the discussions on security and control, the desire for *anonymity* in the e-mentoring platform focused on the potential to allow mentors access to posts in the *forum* and whether forums should be separate for mentees and mentors. The mentors also expressed concerns related to privacy and anonymity of their mentees in the forum. One of the mentors was worried that even in cases where one could post anonymously, the information might be able to be associated with a specific mentee anyway (Multimedia Appendix 1). Anonymity could, thus, lead to a false sense of security and create uncertainty among the users.

#### Messaging

Mentees mentioned potential benefits of sending and receiving documents and similar items (such as a curriculum vitae [CV]) in private messages to mentors. One such benefit was enhancing accountability for doing what was agreed upon, as this gave mentors a way to follow-up (Multimedia Appendix 1). However, there was some debate among the mentees about what information they would want to share with their mentors (as discussed in the theme of security and control). Some of the mentees indicated that they were more comfortable with discussing their own strengths (a component of the nondigitized program) and receiving feedback from mentors online rather than face to face. It was suggested that a digital component could provide a place to initiate discussions of sensitive topics with mentors, building trust by opening the doors for more personal in-person communication. For example, one mentee said she wanted to ask her mentor for advice about sensitive situations, such as how to deal with a fight with a friend and how to resolve problems with her teacher who expressed anti-immigrant sentiments (Multimedia Appendix 1).

Mentors felt that a chat or forum function could be useful in supplementing the face-to-face program by providing a specific arena where they could follow up on tasks and objectives that they and their mentees had agreed upon. One mentor communicated on a weekly basis with his mentee via social media and telephone and felt it benefited the relationship and made it easier to follow up on various goal-oriented tasks (such as writing a job application; Multimedia Appendix 1). This type of follow up could be done via messaging. This mentor noted how his mentee appreciated the opportunity to practice Norwegian language with the mentor on the phone or Facebook and suggested that a messaging function would be similarly useful (Multimedia Appendix 1).

As several mentors expressed concerns about *intruding* in their mentees’ private spaces or social media personas, a dedicated channel for communication (program platform) might be preferable to using social media traditionally used with peers, as suggested by one mentee (Multimedia Appendix 1). This was seen as particularly relevant in cases where mentors experienced challenges in differentiating between mentor and friend roles (Multimedia Appendix 1).

Many of the mentors and mentees were optimistic about the potential for communicating via a messaging function, whereas others did not view this aspect as positively. Mentors expressed concerns about being available at all times for mentee messages and the potential burden this might represent. This specifically concerned messages that might indicate that the mentee was depressed, feeling lonely, or missing their family (Multimedia Appendix 1). Several other mentors felt that they already had sufficient tools for communication (eg, SMS, WhatsApp, and phone) and did not require a new platform for communication with their mentees (Multimedia Appendix 1).

An important issue, which was evident in the discussions with mentors and mentees on the forum and messaging function, was the need for security and control. A suggestion for ensuring privacy (security of data) was to use a stringent log-on (at the same cryptographic level as banking), which almost everyone in Norway has, for access. This would ensure that only people who were part of the program could log on and that family members would not be able to read any personal information (Multimedia Appendix 1).

#### Toolbox

The *toolbox* concept in the original ReConnect platform referred to a wide range of optional interactive support tools and resources. The contents of a toolbox tailored to mentoring activated a variety of ideas among the respondents, ranging from one-way information snippets about the mentoring program to more interactive and individualized support related to the program components.

One activity in the Catalysts program is identifying the youths’ personal strengths, but sometimes it was challenging for mentees to identify and discuss these. It was suggested that a toolbox could support this process by including concrete topics for discussion or allowing mentees to select from predefined categories (Multimedia Appendix 1). Other suggestions for potential components for a toolbox included guidance for getting a job, writing a CV, strengthening Norwegian skills, and similar, thus facilitating goal achievement and connection to Norway. The ability to follow progression in the program generally or progress toward personal goals was also a desirable function (Multimedia Appendix 1). For some of the mentees, mentors were seen as possessing a special kind of knowledge that they wanted to gain access to, both generally and more specifically related to school, the job market, or language. Mentors, in turn, expressed the desire to share their knowledge about their education and career *paths* with mentees or simply assist them in getting work. The toolbox was discussed explicitly as a *super solution* in this context (Multimedia Appendix 1).

Despite receiving information and guidance during the monthly meetings, mentors frequently mentioned needing additional tools during the course of the program. One mentor revealed that he had not used some of the basic components of the program that they learned in training because his mentee did not find them relevant. However, this mentor and others felt that a toolbox could help them concretely understand what tools could be used in practice and, possibly, send them reminders to do so (Multimedia Appendix 1).

By having the platform on a mobile phone, mentors and mentees could also make specific plans for each meeting, addressing the need for information and uncertainty experienced by both parties and providing a knowledge basis with which to *pay it forward*:

Yes, and the toolbox, I was thinking...when the mentor and mentee meet. The first meeting is a bit awkward. So I thought that if the other mentees could write about what they would do with their mentors, then the others could see. So the mentees can learn from...other mentees.

Mentee, Workshop 2

Several mentors viewed the toolbox as a potential *information bank* with practical examples, information on best practices, text or links to various resources, and suggestions on what mentors could do if a challenging situation arose (Multimedia Appendix 1). Such examples could help the mentors to feel more comfortable in their roles and better equipped to deal with uncertainties (Multimedia Appendix 1) as well as better able to help mentees achieve their goals.

#### Additional Components

Through the course of the discussions, additional components for the e-mentoring platform were suggested, such as *calendar* and *GPS* or *position tracking*. Introducing a location-specific calendar could provide an oversight over both program milestones, reminders of program meetings, and activities and events happening in the local community, helping to activate participants and increase the youths’ sense of connection (Multimedia Appendix 1). A shared calendar could also allow mentees to connect with others participating in the program, and it was suggested that mentees could bring along their friends, allowing a *pay it forward* aspect for peers outside of the program. Another proposal was to allow the calendar to be connected to external calendars, so that mentors could check their mentees’ availability (Multimedia Appendix 1). However, some youths found it unnecessary to have a calendar on the digital platform and felt that their own personal calendars were sufficient. Several of the mentees also expressed a desire for tools, such as a GPS, that would help them geographically navigate the local community, especially for meetings in the mentor program or for events (Multimedia Appendix 1). The idea of having a shared *calendar* function was also discussed in light of the need for security and control, with mixed opinions. Some of the mentees wanted the calendar to be private, whereas others felt that they should be open with their mentors (Multimedia Appendix 1).

## Discussion

### Principal Findings

The original pragmatic question posed in this study was about the relevance of an eHealth platform (ReConnect) in the context of a mentoring program (Catalysts). After identifying the key elements of Catalysts’ theory of change as a basis for assessing this question (see the Introduction section), we posed open questions to immigrant youths and mentors about their experiences and needs related to mentoring and potential areas for digital support. The findings are first discussed in light of the literature, irrespective of digital support, followed by implications for digital design and implementation in a mentoring context.

### Mentees and Mentors

Broadly, the experiences and needs expressed by the multicultural youths in this study coincide well with studies that have examined similar groups relative to the concepts of health and social capital. For example, a Canadian study [28] found that refugee youths defined *health* in terms of a sense of belonging, an ability to cope, and self-determination, dimensions closely aligned with the themes identified in this study.

Although the resultant themes were distinct, they, nevertheless, overlapped in certain areas. For example, *security and control* was primarily discussed in the context of digital support issues (protection of personal data), but it tied in with the broader issues of trust and belonging—trust in who the youths communicated with and being part of a community that was safe and supportive. Similarly, the mentees’ need for connection was also reflected in the *goals* theme in that youths sought broader connections to the Norwegian community through their mentors as a means of attaining their goals. These findings are reflective of what others have found [29].

Mentors’ experiences and needs largely reflected their desire to contribute to society by supporting immigrant youths in their development and integration. Mentors saw their role, in part, as sharing their knowledge of how society and its institutions such as school and work functioned. Mentors also expressed a desire for connection and to understand other cultures, as well as enhance their own networks via contact with fellow volunteers. Their reported motivations for becoming a mentor and the insecurities experienced in their roles (eg, how to blend formal [mentor] and informal [friendship] roles) are similar to what others have found [30-34].

#### Implications for Electronic Mentoring Design

Mentees and mentors offered a wide range of ideas and preferences for adapting the main components from the original ReConnect platform (eg, forum, messaging, toolbox) and suggested new components for the e-mentoring platform (such as calendar and GPS). Perhaps the clearest implication of the findings for e-mentoring design, expressed by all informants, is that it must not replace in-person contact between the youths and mentors. Although mentoring that is exclusively based on digital contact may be a *better than nothing* solution for some, informants in this study instead focused on the ways digital support could enhance in-person mentee-mentor encounters and relationships. This echoes the view that the core benefit of mentoring is the *relationship* between the mentee and mentor and concerns that e-mentoring might undermine the dyads’ ability to forge quality relationships [17]. Support for broader networks among mentees and mentors, both individually and collectively, was also emphasized, again as a way of fostering in-person relationships. These findings reflect Catalysts’ own theory of change, highlighting specific areas where e-mentoring could enhance their traditional mentoring in terms of managing the relationships, supporting social capital, and ensuring security.

#### Managing the Relationships

Reflective of the primacy placed on the mentee-mentor relationship, informants’ suggestions for digital resources largely had to do with fostering in-person relationships and activities toward specific goals (relating to main themes 1 and 2). Information that aimed to boost mentor skills and sense of efficacy (eg, case descriptions and various scenarios for responding) as well as mentees’ skills and self-efficacy in eliciting mentor support for their given needs were suggested [35]. Specific tools (forum and toolbox) to support mentee-mentor collaboration around concrete tasks were also important in this regard. For example, many of the suggestions for digital support had to do with tools that could boost specific skills such as Norwegian training, writing a CV, and goal-based planning, along with ways the mentors and mentees could become engaged in developing skills. This ties in with what others have suggested [36]—that specific activities toward concrete goals (*relationship as the context for an intervention*) are preferable to friendship models of mentoring where the main objective is to forge a close bond (with the relationship as the intervention). Although the forum and toolbox were considered valuable for relational support, sending private messages was viewed as less important by most of the stakeholders, as existing tools (eg, SMS and WhatsApp) were largely viewed as sufficient.

#### Social Capital Support

Digital options for facilitating connection and goal orientation (main themes 1 and 2) are further discussed here in the light of social capital [37,38]. Although some discuss digital options for boosting social capital with regard to the plethora of opportunities provided through social media [39], we chose a more limited approach, due to the limited scope of the ReConnect platform to structure the social interactions among the youths and adults.

Informant suggestions for forums varied between those just for mentees, mentors, or combinations, and also in conjunction with offline activities. These permutations have implications in terms of how they might enable or limit different forms of social capital. For example, forums exclusive to mentees were framed as enhancing horizontal bonding types of social capital, that is, connections to other relatively similar youths [23]. At the same time, it was argued that this type of peer support could help youths in navigating and building their relationships with adult mentors. In addition, mentees’ expressed desire to *pay it forward* to other mentees could be facilitated through forum connection and belonging [40]. In contrast, a group forum for mentees and mentors might cultivate bridging social capital, allowing connections across boundaries of ethnicity, age, and social status [41]. Mentees argued that this would considerably expand their access to mentors who may have more relevant skills than their own mentor and are therefore better able to advise in terms of goal achievement.

Arguments for including a shared calendar function showing local events were that it would offer a way for mentees to connect with others and participate in activities that they otherwise might not have prioritized or even been aware of. Some of the youths revealed a desire to increase social connections in their life and extend their networks, whereas for a few, this was viewed as less significant than enhancing the individual relationships they had with their mentors.

Both mentors and mentees wanted the toolbox to provide resources for individual knowledge building and goal achievement and to provide tools to guide the mentor-mentee interactions. Completing activities together in person might be even more effective in strengthening individual-level trust when supported by follow-up options through e-mentoring, again, potentially providing a foundation for more generalized trust. The importance of trust, a central aspect of social capital [18,19], was evident in how mentors and mentees discussed requirements for environments that fostered genuine supportive sharing [17,42].

Building trust across categories of ethnicity, culture, age, and socioeconomic class inevitably requires grappling with challenges of power dynamics and both conscious and unconscious preconceptions [43]. In addition to the training the mentors and mentees receive from the nondigital program, a digital platform may aid in reducing these barriers and their adverse impacts on trust and social capital.

#### Security and Control

Having control over personal information appeared especially important for some of the youths, particularly those who have experienced social control. This was illustrated in the discussions of login processes, where a mentee expressed gratitude for Norway’s strict security requirements compared with their country of origin, as well as in terms of information sharing. On the one hand, as shown in other studies [44], anonymity in a digital social setting can allow information and ideas to flow more freely, for example, by decreasing shyness, and on the other hand, it can also result in the lack of accountability or credibility and deindividuation [44]. Confidentiality is important in mentoring as it may influence the amount of information a mentor or mentee reveals in their relationship [45], which, in turn, contributes to the development of trust [46]. However, previous studies also indicate that confidentiality may be more challenging to maintain in the context of virtual mentoring because users can perceive a false sense of security and disclose more than they might otherwise do face to face [47]. Offline activities, thus, remain critical in enhancing social ties and trust [44], which is again supported by the respondents’ dismissal of replacing in-person contact with an app.

### Limitations and Future Work

Although the Catalysts program endeavors to recruit youths with genuine needs for support, those who participate are self-selected and likely to be more motivated than those who do not show interest for the program. Thus, the participants in this study may have possessed characteristics that distinguish them from the broader population of multicultural youths in Norway. Using ReConnect to facilitate discussions about needs for digital support likely influenced the direction of some interviews. As our point of departure was limited by preparations for an intervention study, which requires a closed and secure environment for social interactions among youths and adults, future research could investigate the benefits and limitations of open social media platforms, compared with more secure and closed environments for mentor-mentee interactions. Also, although not explicitly examined in this study, the individual characteristics of the youths (and adults) in this study (eg, gender, ethnicity, length of time in Norway, and age) can play an important role in their articulated needs and requirements [48]. Future studies would benefit from exploring how these factors influence the reported needs.

### Conclusions

This study investigated mentors’ and mentees’ experiences and needs, providing valuable insights into how to design digital solutions to supplement in-person mentoring of multicultural youth. The desire for connection at the individual and community levels was salient. In addition, a strong emphasis on goal achievement from the stakeholders underscored the motivated character of the youths and the strong desire of mentors to help. Concerns about security and privacy were prevalent in the discussions. Despite the promise of digitalization, mentors and mentees emphasized that the platform should not replace in-person contact. Given attention to the unique needs and preferences of mentoring stakeholders, the features of existing eHealth apps can be adapted to a mentoring program context, potentially enhancing the mentee-mentor relationships and fostering mentee social capital.

## Appendix

Multimedia Appendix 1 Supplementary quotations illustrating themes and subthemes (spreadsheet).

## Abbreviations

CV: curriculum vitae
eHealth: electronic health
e-mentoring: electronic mentoring
NAV: Norwegian Labor and Welfare Administration
NORCE: Norwegian Research Center
